# Tacrolimus Utilization and Expenditure in Serbia

**DOI:** 10.3389/fpubh.2017.00291

**Published:** 2017-11-07

**Authors:** Nemanja Rancic, Neven Vavic, Katarina Obrencevic, Filip Pilipovic, Viktorija Dragojevic-Simic

**Affiliations:** ^1^Centre for Clinical Pharmacology, Medical Faculty of the Military Medical Academy, University of Defence, Belgrade, Serbia; ^2^Solid Organ Transplantation Center, Military Medical Academy, Belgrade, Serbia; ^3^Faculty of Medicine, University of Belgrade, Belgrade, Serbia

**Keywords:** drug utilization, drug expenditure, immunosuppressive agents, tacrolimus, Serbia, solid organ transplantation, pharmacoepidemiology

## Abstract

**Background:**

Increasing immunosuppressant consumption and expenditure is a quite a challenge in transplantation medicine. The aim of the study was to characterize the utilization and expenditure of tacrolimus, backbone, and standard of care in immunosuppression regimen in Serbian solid organ transplant recipients.

**Methods:**

This study was performed as retrospective cross-sectional study during a 3-year period (from 2013 to 2015) in Serbia. The Anatomical Therapeutic Chemical Classification/Defined Daily Doses (ATC/DDD) international system was used for consumption evaluation.

**Results:**

Two hundred and sixty-nine patients were transplanted in Serbia from 2013 to 2015 (185 recipients from deceased donors and 84 recipients from living donors). Total number of deceased donors in this period was 81. The consumption of tacrolimus increased (from 0.051 DDD/1,000 inhabitants/day to 0.069 DDD/1,000 inhabitants/day in 2013 and 2015, respectively). The total cost of tacrolimus was also increased; from 1,206,816€ to 1,483,472€ in 2013 and 2015, respectively. On the other hand, the number of all new solid organ transplants (from deceased and living donors) per million population per year was decreased from 17.39 to 10.02, from 2013 to 2015, respectively.

**Conclusion:**

In spite downward trend in the number of solid organ transplants, tacrolimus consumption and expenditure in the examined 3-year period in Serbia increased. Since tacrolimus is a high-cost and life-preserving drug, its increasing utilization and expenditure will most likely continue consuming an enhancing share of Serbian pharmaceutical expenditure, as well as its health care, as a whole.

## Introduction

Nowadays, solid organ transplantation is considered the treatment of choice for many people with severe chronic diseases, but still there is a shortage of organs available for donation (1). Moreover, the costs of organ transplantation include transplant evaluation and testing, transplant surgery, follow-up care, and medication. The average reported cost of solid organ transplant ranges from $260,000 for a single kidney transplant to over $1.2 million dollars for combined heart and lung transplants (2).

One of the greatest challenges facing the field of organ transplantation today is continuous increasing of immunosuppressant utilization and expenditure (3). Since it is well known that immunosuppression is required for the lifetime of a solid organ transplant to prevent rejection, the cost of these medications is constant concern not only for health insurance funds but especially for those lacking long-term insurance coverage (4, 5).

Not only oral immunosuppressive agents and other necessary drugs but also insurance expenses can cost patients up to $2,500.00 per month, depending of patient clinical condition and other factors (5). In the United States, annual cost of medications accounts between $10,000 and $14,000 per patient (6).

Induction therapy is given at the time of transplantation, while later on various combination of orally taken agents are necessary. Current recommended protocols in the patients subjected to solid organ transplantation include calcineurin inhibitors (tacrolimus or cyclosporine) and antiproliferative agents (mycophenolate mofetil or azathioprine), with or without regimens of corticosteroids (4, 7, 8).

Tacrolimus is the first-line treatment according to the Kidney Disease Improving Global Outcomes Transplant Work Group (4) and is also commonly used in the liver and heart transplantation (3, 9). It was first developed as a twice-daily oral formulation (tacrolimus-BID), while once-daily tacrolimus (tacrolimus-OD) appeared in order to improve drug compliance and lower pill burden (10). It was shown that tacrolimus-OD ensure better drug compliance comparing with tacrolimus-BID, associated with improved graft outcome, glucose tolerance, and lipid profile (11, 12).

In our previous study, it was shown that immunosuppressive drugs are significant factor of the total cost of organ transplantation (13). Similar results were obtained in some other studies (3, 14–16).

Since tacrolimus has been the cornerstone of the standard immunosuppressive regimen in solid organ transplant recipients, the aim of our study was to characterize its utilization and expenditure in Serbian population of patients.

## Materials and Methods

Retrospective cross-sectional study was performed during a 3-year period (from 2013 to 2015). Anatomical Therapeutic Chemical Classification/Defined Daily Doses (ATC/DDD) international system for classification and consumption of drugs according to the WHO methodology was used for tacrolimus consumption (17). In the ATC classification, drugs are assorted into different groups in accordance with their mechanism of action and according to the therapeutic, pharmacological, and chemical properties. DDD values are the average maintenance dose per day for a drug when it is applied in its main indication, concerning average adult. DDD per 1,000 inhabitants/day is a measurement unit used for tacrolimus consumption. DDD used for oral application of tacrolimus was 5 mg, while ATC code was L04AD02. Calculations were done according the following equation:

$$\text{DDD}/1,000\text{ inhabitans}/\text{day}=\frac{\text{Amount of drug }(\text{mg})\text{ sold in 1 year}}{\text{DDD}(\text{mg})\times 365\text{ days}\times \text{Number of people}}\times 1,000.$$

Tacrolimus utilization and expenditure in Serbia is based on official data released by the Medicines and medical devices agency of Serbia and national Health insurance fund (18). In accordance with the Law on Medicines in Serbia, Medicines and medical devices agency of Serbia collects and processes data on marketing and consumption of medicinal products in our country and publishes them annually. These publications contain health, economic, and statistical indicators concerning medicinal product use in Serbia.

On the other hand, data concerning the number of renal, liver, and heart organ transplantations, number of deceased/living donors, and the number of all new solid organ transplants from deceased/living donors per million population per year are based on the data obtained from Department of Biomedicine, Ministry of Health, Republic of Serbia (19). The number of total people in Serbia was 7,186,862, according to latest census of population in 2011 (20).

Microsoft Office Excel 2007 was used for statistical analysis. Trend analysis was used for data processing concerning observed period from 2013 to 2015.

### Ethical Approval

The principles of ICH Good Clinical Practice were strictly followed and ethical approval No. 01/31-01-13 from the Ethics Committee of the Military Medical Academy was obtained for the study protocol No. 910-1.

## Results

**Figure 1:**
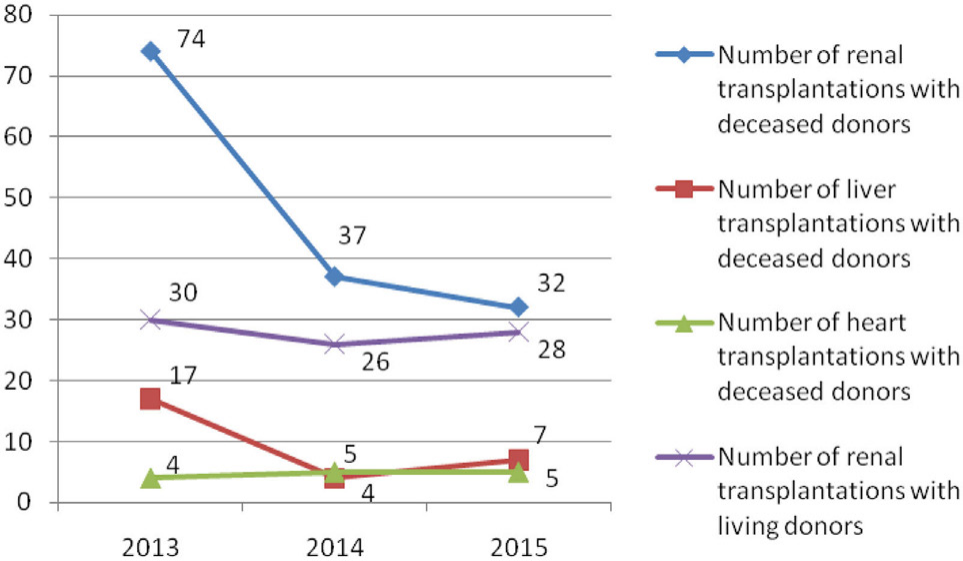
Number of renal, liver, and heart organ transplantations in Serbia from 2013 to 2015.

**Figure 2:**
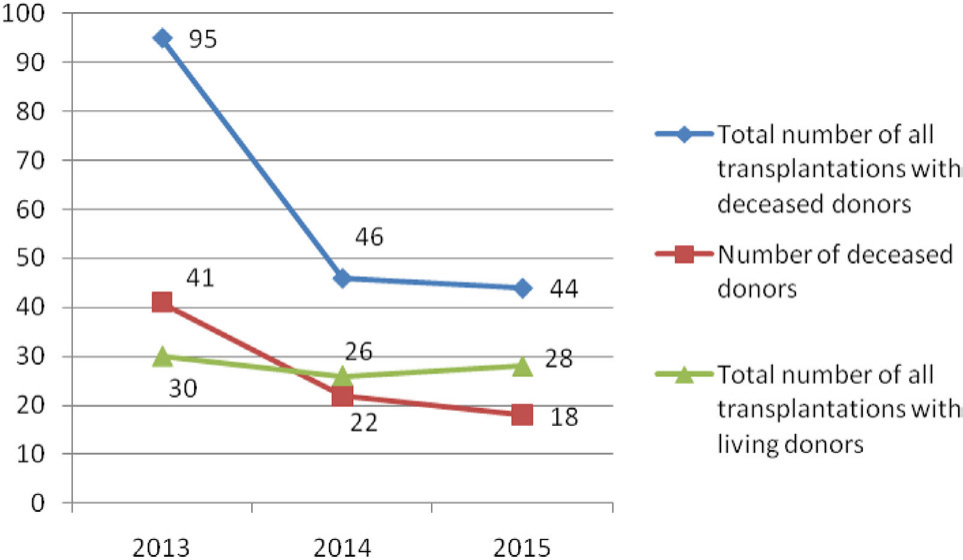
Number of total solid organ transplantations and number of donors in Serbia during the examined 3-year period.

During the observed 3-year period, 269 patients were transplanted (185 recipients with deceased donors and 84 recipients with living donors) (Figures 1 and 2). The most of transplant patients received a renal cadaveric graft (74, 40, and 11 in 2013, 2014, and 2015, respectively). Total number of cadaveric donors was 41, 22, and 18 in 2013, 2014, and 2015, respectively.

**Figure 3:**
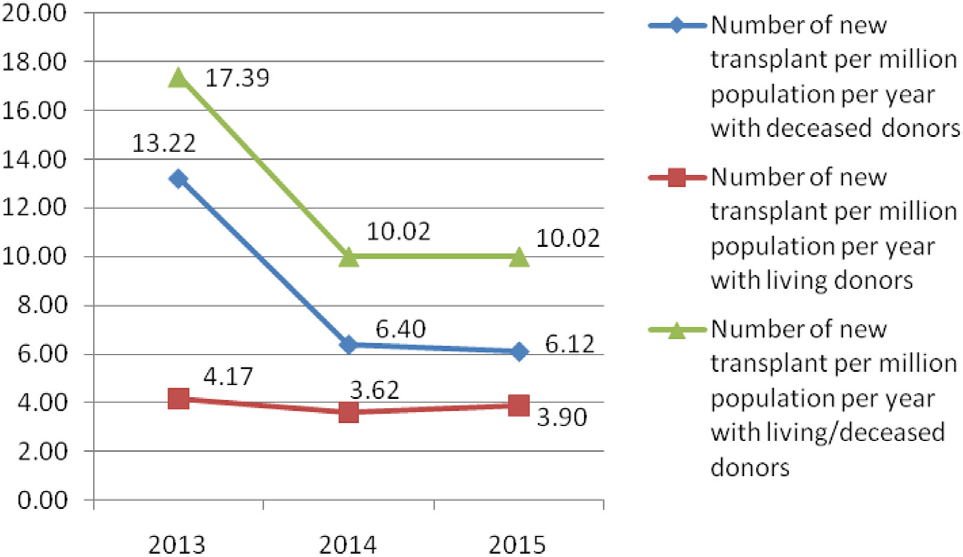
Number of all solid organ transplant per million population per year in Serbia from 2013 to 2015.

In Serbia, the number of all new solid organ transplants with deceased donors per million population per year continually decreased from 13.22 to 6.12 during the 3-year period (2013–2015) (Figure 3). The number of new solid organ transplants with living donors per million population per year was similar during the 3-year period (from 4.17 to 3.90, in 2013 and 2015, respectively).

**Figure 4:**
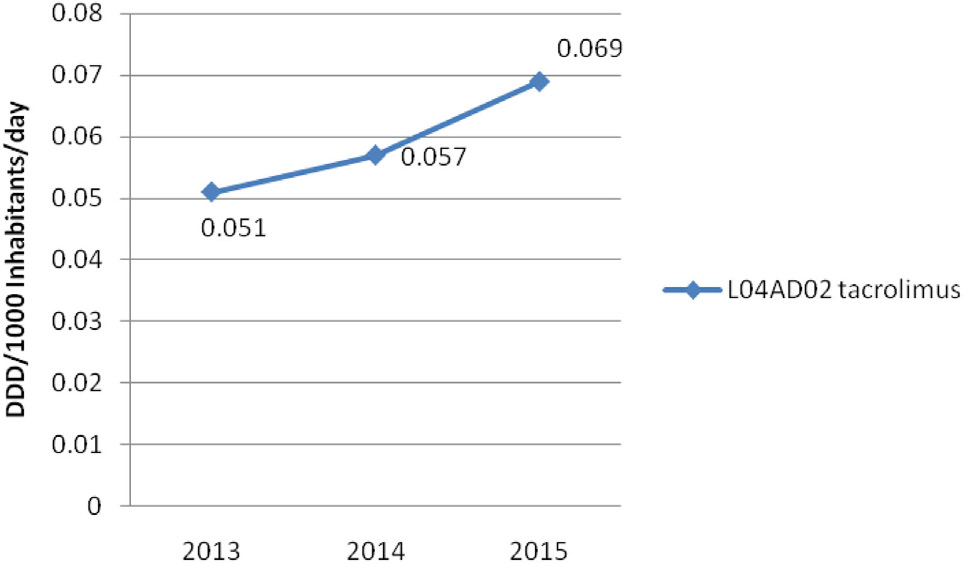
Utilization of tacrolimus in Serbia in the period from 2013 to 2015.

**Figure 5:**
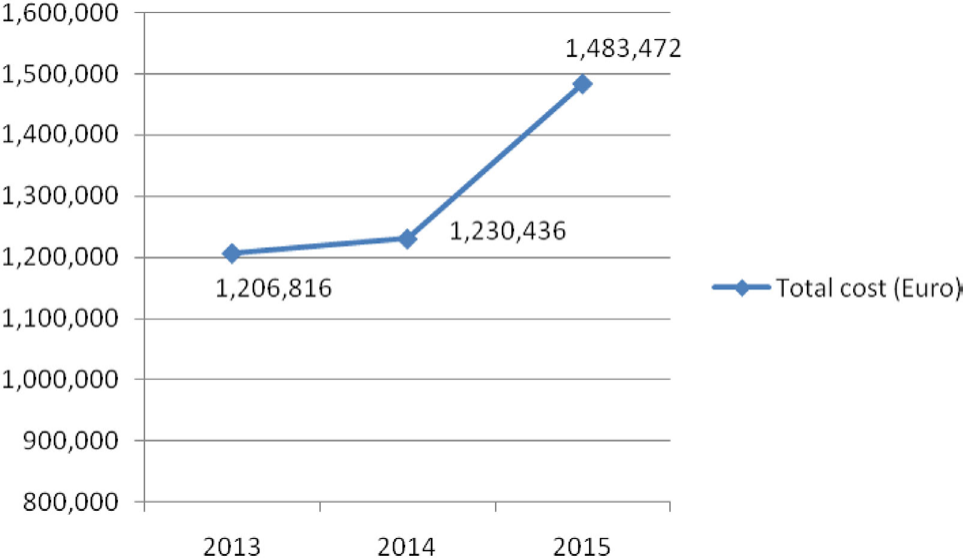
Total cost of tacrolimus in Serbia during the examined 3-year period.

On the other hand, Figure 4 shows consumed amounts of tacrolimus based on DDD and DDD/1,000 inhabitants per day units during examined 3-year period. The consumption of tacrolimus was constantly increasing (from 0.051 DDD/1,000 inhabitants per day to 0.069 DDD/1,000 inhabitants per day; in 2013 and 2015, respectively). Also, the total cost of tacrolimus was constantly increasing (Figure 5); from 2013 to 2015 total cost was increased for 22.92%, i.e., from 1,206,816€ to 1,483,472€.

## Discussion

The public spending on pharmaceuticals in Serbia was doubled in less than a decade (from 2004 to 2012) (21–23). This growth was recorded mostly due to expenditure of statins, novel platelet aggregation inhibitors, monoclonal antibodies, combined preparations indicated in asthma, and chronic obstructive pulmonary disease, as well as immunosuppressive drugs like tacrolimus. Therefore, the main working hypothesis of our study was the positive trend in the consumption of tacrolimus in our country. According to that the aim of our study was to characterize its utilization, as well as expenditure in Serbian population of patients during the observed 3-year period, from 2013 to 2015.

Utilization of tacrolimus in Australian population was 0.248 DDD/1,000 inhabitants per day in 2013 (3). The total consumption of tacrolimus in Serbia was equal to 20.56% of the consumption of tacrolimus in Australia population in the same year. On the other hand, in year 2012 number of new renal transplants per million population per year was about 55 in Australian population (3), whereas in our country it accounted for 13.22 for all solid organ transplantation in year 2013. The number of donors in our country was continually decreasing.

However, number of patients on waiting lists, not only in our country, reflects difficult situation in lacking the suitable organ, concerning various transplant systems with different national policies (24). In Europe, if patients from Iceland, Norway, and Turkey are added, 86,000 patients were on the waiting lists in 2013 (for a total population of 588 million inhabitants) (24). However, many patients die while officially have been placed on these waiting lists. Existence of the European organ exchange organizations (Eurotransplant, Scandiatransplant, and South Alliance for Transplants) is very important for increasing the number of donors (24, 25). Serbia is in the phase of accession to the Eurotransplant, what should reduce waiting lists and increase the number of donors and transplants realized.

Renal transplantation is most often performed in comparison to other solid organ transplants (liver, heart) (3). Costs per one transplanted patient in Serbia for a 10-year period were calculated to be €48,949 (26). Its Gross Domestic Product (GDP) per capita is $5,348.29 in 2016 year. Although their GDP/capita is not so high comparing with other countries from the Western World ($12,090.66 in 2016 comparing with 15,893.86 in 2008), Croatian health-care system affords costly procedures such a kidney transplants: their number of 50 transplants per million population is among the top countries of Europe (27). Therefore, the performance of the countries such as Croatia shows that GDP per capita need not to be a dominating factor in the successful solid organ transplantation. According to this report (27), Serbia is on the last place by the number of renal transplants per million population per year in 2016.

As it was mentioned, the cost of immunosuppressive drugs is constant concern since they are required for the lifetime in patients subjected to solid organ transplantation. According to the data of US United Network for Organ Sharing, costs are more than $262,000 just for the first year after kidney transplantation (28). The total monthly costs of Cellcept^®^, Prograf^®^, Prednisone^®^, and Myfortic^®^ were $1,064, $1,340, $12, and $806, respectively. The 2-year costs of four different immunosuppressive drugs (sirolimus, everolimus, cyclosporine, or tacrolimus) have been shown to vary between €26,732 and €49,978 (29). In Iran, the total cost of renal transplantation procedure was $9,224, while the immunosuppressive therapy accounted for even 65.8%, only in the first year (30).

In Croatia, the total cost of tacrolimus was 2,156,521€ in 2013; 1,942,018€ in 2014; and 2,449,061€ in 2015. The consumption of tacrolimus was higher in the same period than in Serbia (0.19, 0.15, and 0.18 DDD/1,000 inhabitants per day; in 2013, 2014, and 2015, respectively) (31). Therefore, in 2015, the consumption of tacrolimus was 2.6-fold higher in Croatia comparing with Serbia, while its expenditure was 1.65-fold higher in Croatia in comparison to our country.

The cost and consequences of medication non-adherence should also be taken into account when the price of immunosuppression and of solid organ transplantation, as a whole, are considered (32). The introduction to the market of generic formulations will probably decrease the financial burden concerning immunosuppresive agents. However, costs concerning conversion, as well as initial expenses due to laboratory monitoring should be taken into account (33). Other costs that should be included are those concerning adverse effects, episodes of rejection, etc. The fact that there is relatively small market of transplantation can also explain escalating costs of therapy. Therefore, although the number of all new solid organ transplants per million population per year continually decreased in Serbia, the prevalence rate of the transplantation is constantly increasing due to the fact that transplant recipients’ lifespan and long-term allograft survival are improved. Also, in our country transplantologists also take care of the transplant recipients who were subjected to transplantation in other countries. All of this leads to expectations that tacrolimus utilization and expenditure will most likely to continue to grow.

The limitation of the study is that we have not been able to evaluate the trend in the consumption of other immunosuppressants used in solid organ transplantation in our country (azathioprine, corticosteroids, cyclosporine, sirolimus, everolimus, mycophenolic acid, biologicals) and compared it with tacrolimus. Namely, these drugs have numerous other indications and it has not been possible, due to the nature of the available data concerning their consumption, to include them in this study. However, it is highly recommended that Cost-Effectiveness Analysis studies between different immunosuppressive drugs used in Serbian solid organ transplant recipients would be performed in the future.

## Conclusion

In spite downward trend in the number of solid organ transplants, tacrolimus consumption and expenditure increased in the examined period from 2013 to 2015 in Serbia. Since tacrolimus is a high-cost and life-preserving drug, its increasing utilization and expenditure will most likely continue consuming an enhancing share of Serbian pharmaceutical expenditure, as well as its health care, as a whole.

## Author Contributions

All the authors (NR, NV, KO, FP, and VD-S) listed have made substantial contribution to the conception, design, analysis, and interpretation of data for the work and approved it for publication.

## Conflict of Interest Statement

The authors declare that the research was conducted in the absence of any commercial or financial relationships that could be construed as a potential conflict of interest.

## References

[1] Grinyó JM. Why is organ transplantation clinically important? Cold Spring Harb Perspect Med (2013) 3(6):a014985. 10.1101/cshperspect.a014985 23732857 PMC3662355

[2] Bentley TS Hanson SG 2011 U.S. Organ and Tissue Transplant Cost Estimates and Discussion. Brookfield, WI: Milliman (2011). 20 p.

[3] Gardiner KM Tett SE Staatz CE. Multinational evaluation of mycophenolic acid, tacrolimus, cyclosporin, sirolimus, and everolimus utilization. Ann Transplant (2016) 21:1–11. 10.12659/AOT.895664 26729299

[4] Kidney Disease: Improving Global Outcomes (KDIGO) Transplant Work Group. KDIGO clinical practice guideline for the care of kidney transplant recipients. Am J Transplant (2009) 9(Suppl 3):S1–155. 10.1111/j.1600-6143.2009.02834.x 19845597

[5] James A Mannon RB. The cost of transplant immunosuppressant therapy: is this sustainable? Curr Trans Rep (2015) 2(2):113–21. 10.1007/s40472-015-0052-y 26236578 PMC4520417

[6] Kasiske BL Cohen D Lucey MR Neylan JF. Payment for immunosuppression after organ transplantation. American Society of Transplantation. JAMA (2000) 283(18):24445–50. 10.1001/jama.283.18.2445 10815094

[7] Birdwell K. Role of pharmacogenomics in dialysis and transplantation. Curr Opin Nephrol Hypertens (2014) 23(6):570–7. 10.1097/MNH.0000000000000065 25162201 PMC4220684

[8] Karam S Wali RK Current state of immunosuppression: past, present, and future. Crit Rev Eukaryot Gene Expr (2015) 25(2):113–34. 10.1615/CritRevEukaryotGeneExpr.2015011421 26080606

[9] Vavic N Rancic N Dragojevic-Simic V Draskovic-Pavlovic B Bokonjic D Ignjatovic L The influence of comedication on tacrolimus blood concentration in patients subjected to kidney transplantation: a retrospective study. Eur J Drug Metab Pharmacokinet (2014) 39(4):243–53. 10.1007/s13318-013-0168-3 24356808

[10] Staatz CE Tett SE Clinical pharmacokinetics of once-daily tacrolimus in solid-organ transplant patients. Clin Pharmacokinet (2015) 54(10):993–1025. 10.1007/s40262-015-0282-2 26038096

[11] Uchida J Kuwabara N Machida Y Iwai T Naganuma T Kumada N Conversion of stable kidney transplant recipients from a twice-daily Prograf to once-daily tacrolimus formulation: a short term study on its effect on glucose metabolism. Transplant Proc (2012) 44(1):128–33. 10.1016/j.transproceed.2011.11.005 22310596

[12] Tsuchiya T Ishida K Oto S Deguchi T. Effect of conversion from twice-daily to once-daily tacrolimus on glucose intolerance in stable kidney transplant recipients. Transplant Proc (2012) 44(1):118–20. 10.1016/j.transproceed.2011.11.027 22310593

[13] Rancic N Dragojevic-Simic V Vavic N Kovacevic A Segrt Z Djordjevic N. Economic evaluation of pharmacogenetic tests in patients subjected to renal transplantation: a review of literature. Front Public Health (2016) 4:189. 10.3389/fpubh.2016.00189 27630984 PMC5005394

[14] Lee EK Tseng PL. Retrospective study on the utilization and cost of immunosuppressive agents among kidney transplant recipients in Taiwan: a 5-year review. Transplant Proc (2008) 40(7):2214–7. 10.1016/j.transproceed.2008.07.003 18790196

[15] Lee EK Cham TM Tseng PL A retrospective study on the utilization of and expenditure for immunosuppressants for organ transplant recipients in Taiwan – updated to 2006. Transplant Proc (2010) 42(3):961–5. 10.1016/j.transproceed.2010.03.013 20430216

[16] Lee EK Cham TM Tseng PL. Using the pharmacoepidemiology approach to evaluate the first-year posttransplantation ambulatory health care cost from the longitudinal health insurance database (2001 to 2006) in Taiwan. Transplant Proc (2010) 42(3):957–60. 10.1016/j.transproceed.2010.03.011 20430215

[17] WHO Collaborating Centre for Drug Statistics Methodology. Definition and General Consideration (2013). Available from: https://www.whocc.no/filearchive/publications/1_2013guidelines.pdf

[18] Medicines and medical devices agency of Serbia. Marketing and Consumption of Medicinal Products in Serbia (2017). Available from: https://www.alims.gov.rs/ciril/o-agenciji/publikacije/

[19] Ministry of Health, Republic of Serbia. Department of Biomedicine. (2017). Available from: http://www.zdravlje.gov.rs/showpage.php?id=286

[20] Statistical Office of the Republic of Serbia. Population (2017). Available from: http://webrzs.stat.gov.rs/WebSite/Default.aspx

[21] Jakovljevic MB Djordjevic N Jurisevic M Jankovic S. Evolution of the Serbian pharmaceutical market alongside socioeconomic transition. Expert Rev Pharmacoecon Outcomes Res (2015) 15(3):521–30. 10.1586/14737167.2015.1003044 25592856

[22] Pejcic AV Jakovljevic M Pharmaceutical expenditure dynamics in the Balkan countries. J Med Econ (2017) 20(10):1013–7. 10.1080/13696998.2017.1333514 28532192

[23] Jakovljevic MB Souliotis K Pharmaceutical expenditure changes in Serbia and Greece during the global economic recession. SEEJPH (2016) V:49–62. 10.4119/UNIBI/SEEJPH-2016-101

[24] European Commission. Journalist Workshop on Organ Donation and Transplantation. Recent Facts & Figures (2014). Available from: http://ec.europa.eu/health/sites/health/files/blood_tissues_organs/docs/ev_20141126_factsfigures_en.pdf

[25] Spasovski G Busic M Pipero P Sarajlić L Popović AS Dzhaleva T Current status of transplantation and organ donation in the Balkans – could it be improved through the South-eastern Europe Health Network (SEEHN) initiative? Nephrol Dial Transplant (2012) 27(4):1319–23. 10.1093/ndt/gfs071 22467749

[26] Perović S Janković S. Renal transplantation vs. hemodialysis: cost-effectiveness analysis. Vojnosanit Pregl (2009) 66(8):639–44. 10.2298/VSP0908639P 19780419

[27] Health Consumer Powerhouse. Euro Health Consumer Index 2016. Report (2017). Available from: http://www.healthpowerhouse.com/publications/euro-health-consumer-index-2016/

[28] California Pacific Medical Center, Part of the Sutter Health Network. Costs and Financing of Kidney Transplant (2014). Available from: http://www.cpmc.org/advanced/kidney/patients/topics/financing_kidney_transplant.html

[29] Woodroffe R Yao GL Meads C Bayliss S Ready A Raftery J Clinical and cost-effectiveness of newer immunosuppressive regimens in renal transplantation: a systematic review and modelling study. Health Technol Assess (2005) 9(21):1–179. 10.3310/hta9210 15899149

[30] Nourbala MH Einollahi B Kardavani B Khoddami-Vishte HR Assari S Mahdavi-Mazdeh M The cost of kidney transplantation in Iran. Transplant Proc (2007) 39(4):927–9. 10.1016/j.transproceed.2007.03.056 17524852

[31] Agency for Medicinal Products and Medical Devices of Croatia HALMED. Distribution, Manufacturing and Inspection: Drug Utilisation Reports (2017). Available from: http://www.halmed.hr/en/Promet-proizvodnja-i-inspekcija/Promet/Potrosnja-lijekova/Izvjesca-o-prometu-lijekova/

[32] Evans RW Applegate WH Briscoe DM Cohen DJ Rorick CC Murphy BT Cost-related immunosuppressive medication nonadherence among kidney transplant recipients. Clin J Am Soc Nephrol (2010) 5(12):2323–8. 10.2215/CJN.04220510 20847093 PMC2994095

[33] Enspr CR Trofe-Clark J Gabardi S Mcdevitt-Potter LM Shulio MA Genetic maintenance immunosuppression in solid organ transplant recipients. Pharmacotherapy (2011) 31(11):1111–29. 10.1592/phco.31.11.1111 22026398

